# Sperm calcium flux and membrane potential hyperpolarization observed in the Mexican big-eared bat *Corynorhinus mexicanus*

**DOI:** 10.1242/jeb.244878

**Published:** 2023-01-30

**Authors:** José Edwin Mendoza-Sánchez, Ahiezer Rodríguez-Tobón, Edith Arenas-Ríos, Gerardo J. Orta-Salazar, Miguel A. León-Galván, Claudia L. Treviño Santa Cruz, Julio C. Chávez

**Affiliations:** ^1^ Doctorado en Biología Experimental, Universidad Autónoma Metropolitana, Iztapalapa, 09310 Ciudad de México, México; ^2^ Laboratorio de Biología y Ecología de Mamíferos, Universidad Autónoma Metropolitana, Iztapalapa, 09310 Ciudad de México, México; ^3^ Laboratorio de Morfofisiología y Bioquímica del Espermatozoide, Universidad Autónoma Metropolitana, Iztapalapa, 09310 Ciudad de México, México; ^4^ Consorcio de Fisiología del Espermatozoide, Instituto de Biotecnología, Universidad Nacional Autónoma de México, Cuernavaca, 62210 Morelos, México

**Keywords:** CatSper, Sperm storage, Ion channel, Capacitation

## Abstract

Mammalian sperm capacitation involves biochemical and physiological changes, such as an increase in intracellular calcium ion concentration ([Ca^2+^]_i_), hyperpolarization of the plasma membrane potential and sperm hyperactivation, among others. These changes provide sperm with the ability to fertilize. In the bat *Corynorhinus mexicanus*, there is an asynchrony between spermatogenesis and sperm storage in the male with the receptivity of the female. For instance, in *C. mexicanus*, spermatogenesis occurs before the reproductive season. During the reproductive period, sperm are stored in the epididymis for a few months and the testis undergoes a regression, indicating low or almost null sperm production. Therefore, it is unclear whether the elements necessary for sperm fertilization success undergo maturation or preparation during epididymis storage. Here, we characterized pH-sensitive motility hyperactivation and Ca^2+^ influx in sperm, regulated by alkalinization and progesterone. In addition, by electrophysiological recordings, we registered currents that were stimulated by alkalinization and inhibited by RU1968 (a CatSper-specific inhibitor), strongly suggesting that these currents were evoked via CatSper, a sperm Ca^2+^-specific channel indispensable for mammalian fertilization. We also found hyperpolarization of the membrane potential, such as in other mammalian species, which increased according to the month of capture, reaching the biggest hyperpolarization during the mating season. In conclusion, our results suggest that *C. mexicanus* sperm have functional CatSper and undergo a capacitation-like process such as in other mammals, particularly Ca^2+^ influx and membrane potential hyperpolarization.

## Teaser

**Summary:** CatSper activity in bat spermatozoa, as well as events during capacitation, such as hyperactivation and plasma membrane hyperpolarization, correlate with sperm maturation during storage in the epididymis.

**Abbreviations**

4-AP: 4-aminopyridine
AFU: arbitrary fluorescence unit
ALH: amplitude of lateral head movement
ANOVA: analysis of variance
BWW: Biggers, Whitten and Whittingham
Ca^2+^: calcium ion
CAP: capacitated
CatSper: sperm-specific cation channel
*E*_m_: membrane potential
HCO_3_^−^: bicarbonate
K^+^: potassium ion
KO: knockout
LIN: linearity
NC: non-capacitated
P4: progesterone
pH_i_: intracellular hydrogen potential
SEMARNAT: Secretaría de Medio Ambiente y Recursos Naturales
UNAM: Universidad Nacional Autónoma de México
VCL: curvilinear velocity

## INTRODUCTION

The physiology of spermatozoa and the fertilization process have been studied in various animal models; however, in Chiroptera, knowledge is limited. Some species of bats experience physiological lethargy during the winter season, triggering reproductive inactivity during that period. However, a group of species have incorporated interesting reproductive strategies into their life cycle, which allow them to manage their energy to deal with unfavorable environmental conditions and achieve reproductive success. *Corynorhinus mexicanus* is a vespertilionid bat, characterized by its big ears. It is an insectivorous bat, endemic to Mexico, with distribution in regions including the Sierra Madre Oriental, the Transverse Volcanic Axis and the Sierra Madre Occidental, typically found in high, humid mountainous areas dominated by pine–oak forest (Koopman, 1974; López-Wilchis, 1989; Tumlison, 1992). This species presents an asynchronous seasonal monoestrous reproductive pattern (León-Galván et al., 2005; Cervantes et al., 2008; Rodríguez-Tobón et al., 2016). In this bat, spermatogenesis occurs from May to September, but maximum development of the accessory glands and copulation take place in October to December. Therefore, sperm are stored for 5–6 months in the epididymal cauda, which allows males to synchronize with the arrival of receptive females (León-Galván et al., 2005). In the case of *C. mexicanus* females, they present delayed fertilization because they remain in an anovulatory condition and store sperm in the reproductive tract until the release of the oocyte at the end of January (León-Galván et al., 1999). This strategy allows them to give birth in the period of the year when environmental conditions are suitable for optimal development of the offspring. Altogether, prolonged sperm storage in *C. mexicanus* is estimated at approximately 5 months between their permanence in the male and female reproductive tracts, a phenomenon that should have implications for the sperm capacitation process. It has been shown in *C. mexicanus* that sperm maintain their structural and functional integrity during the prolonged storage in the epididymis without losing their fertilizing potential (Cervantes et al., 2008; Rodríguez-Tobón et al., 2016). Also, the presence of cytoplasmic droplets in the spermatozoa decreases according to the time of storage in the epididymis, with most cells with droplets in the first month (September) of epididymal storage and almost no cells with cytoplasmic droplets in the last month of epididymal storage (November) (Cervantes et al., 2008). Additionally, the occurrence of acrosomal exocytosis (using the Coomassie Brilliant Blue method) in spermatozoa collected in November is higher than that in spermatozoa collected in September, indicating a greater maturation process in the last months of epididymal storage (Cervantes et al., 2008). Rodríguez-Tobón et al. (2016) carried out sperm *in vitro* capacitation using a chlortetracycline (CTC) procedure, determining that the percentage of capacitated cells increases with the storage time of the sperm in the epididymal cauda. In addition, tyrosine residue phosphorylation shows the highest level at the beginning of epididymal storage, indicating the activation of flagellar proteins for the acquisition of progressive motility (Rodríguez-Tobón et al., 2016). Although the percentage of capacitated cells has been analyzed with CTC, it is still unknown which biochemical and physiological changes are associated with spermatozoa maturation in *C. mexicanus*, in part because the genome of this bat has not been sequenced. Therefore, in this study, we investigate two factors: (1) whether in *C. mexicanus* similar processes occur as in other mammalian species that allow capacitation, such as hyperactivation, the presence of molecular entities such as CatSper and the occurrence of plasma membrane hyperpolarization; and (2) whether spermatozoa storage in the epididymis is important for activation of the elements that allow the capacitation of spermatozoa once inseminated within the female reproductive tract.

## MATERIALS AND METHODS

### Reagents and solutions

We employed Biggers, Whitten and Whittingham (BWW) medium (Biggers et al., 1971) (95 mmol l^−1^ NaCl, 5 mmol l^−1^ KCl, 1.7 mmol l^−1^ CaCl_2_, 1.1 mmol l^−1^ KH_2_PO_4_, 1.19 mmol l^−1^ MgSO_4_ – 7H_2_O, 25.07 mmol l^−1^ NaHCO_3_, 10 mmol l^−1^ HEPES and 5.56 mmol l^−1^ d-glucose) for non-capacitated (NC) conditions. For capacitated (CAP) conditions, we used BWW-supplemented [1.19 mmol l^−1^ sodium pyruvate, 21.58 mmol l^−1^ Na^+^ lactate and 0.1% mmol l^−1^ bovine serum albumin (BSA; w/v)]. For electrophysiological recordings, we employed hypertonic saline (HS) physiological solution (135 mmol l^−1^ NaCl, 5 mmol l^−1^ KCl, 1.8 mmol l^−1^ CaCl_2_, 1 mmol l^−1^ MgSO_4_, 10 mmol l^−1^ lactic acid, 1 mmol l^−1^ sodium pyruvate, 5 mmol l^−1^ glucose, 20 mmol l^−1^ HEPES) and divalent cation-free (DVF) solution (150 mmol l^−1^ sodium gluconate, 2 mmol l^−1^ EDTA-Na^+^, 2 mmol l^−1^ EGTA, 20 mmol l^−1^ HEPES).

The following reagents were purchased from Sigma-Aldrich (St Louis, MO, USA): valinomycin, carbonyl cyanide m-chlorophenylhydrazone (CCCP), ionomycin, ammonium chloride (NH_4_Cl) and progesterone (P4). 3,3'-Dipropylthiadicarbocyanine iodide [DiSC3-(5)] was obtained from Invitrogen (Carlsbad, CA, USA). Fluo-3 acetoxymethyl ester (Fluo-3-AM) was purchased from Life Technologies Corporation (Invitrogen).

### Capture of animals

The Mexican big-eared bat (*Corynorhinus mexicanus* G. M. Allen 1916) is not included in any category of endangered animals according to the NORMA Oficial Mexicana (NOM-059-SEMARNAT-2010). The specimens used in the study were collected under government authorization number SGPA/DGVS/07397/19, granted to A.R.-T. by the General Directorate of Wildlife, Secretaría de Medio Ambiente y Recursos Naturales (SEMARNAT) agency.

The specimens were captured during the day using an expanded hand net (Bioquip Tropic net). This net is considered one of the most appropriate capture methods for bats when they are in a dormant condition (Kurta y Kunz, 1988). The captures were made inside the refuge called ‘El Túnel’, located at 19°37′14″ N, 98°02′02″ W; 3320 m altitude, in the municipality of Tlaxco, Tlaxcala, Mexico. One capture was done monthly during the stage of the reproductive cycle in which sperm are stored in the cauda of the epididymis of *C. mexicanus* (September to November).

The individuals were selected based on the criteria of complete closure of the metacarpophalangeal epiphyses of the fourth finger (Kunz and Anthony, 1982) and macroscopic characteristics of the epididymis, which is scrotal and elongated, with a bulbous appearance and a whitish tone, indicative of sexual maturity and presence of sperm (León-Galván et al., 2005; Rodríguez-Tobón, 2016). The bats were transported individually and alive in blanket sacks to the laboratory of the Consorcio de Fisiología del Espermatozoide, Departamento de Genética del Desarrollo y Fisiología Molecular in the Instituto de Biotecnología, Universidad Nacional Autónoma de México (UNAM), Cuernavaca, Morelos, México. The capture, revision and handling of the specimens was carried out by the person in the group with sufficient training and experience in the management of the species, who is part of the permanent research program on the reproductive biology of bats in Mexico. Therefore, bat manipulation was in strict adherence with the standard methods of bat management indicated in the main guidelines on the care and use of wildlife (Sikes and Gannon, 2011).

### Sperm collection

The bats were not anesthetized because the effect of the anesthetic could influence the physiology of their sperm. Therefore, the animals were decapitated by highly trained personnel. The procedure employed was approved by the Universidad Autónoma Metropolitana (UAM) bioethics committee. The epididymis and testicles were removed and weighed. Only the caudal region of the epididymis was used to obtain motile sperm, using the swim-out technique (León-Galván et al., 2005; Rodríguez-Tobón, 2016; World Health Organization, 2010). Briefly, two cuts were made in the cauda with dissection scissors, and the tissue was placed in an Eppendorf tube with 1 ml BWW medium (37°C, pH 7.2) for 30 min to allow sperm to swim out.

### *In vitro* sperm capacitation

The sperm sample was divided into two aliquots of ∼90×10^6^ sperm ml^−1^ (NC and CAP) and stored for 6 h at pH 7.2, 37°C, 5% CO_2_ (Cervantes et al., 2008; Rodríguez-Tobón et al., 2016).

### Single-cell intracellular Ca^2+^ concentration ([Ca^2+^]_i_) assays

From the NC and CAP sperm samples, aliquots of 5×10^6^ sperm ml^−1^ were taken and incubated with Fluo-3-AM (2 µmol l^−1^) for 30 min (37°C, 5% CO_2_) protected from light (Mata-Martínez et al., 2018). Subsequently, each sample was centrifuged (300 ***g***, 5 min), and the supernatant was removed. The sample was resuspended in BWW medium for NC conditions (pH 7.2), whereas the CAP sample was resuspended in 200 µl BWW medium supplemented with HCO_3_^−^ and BSA (pH 7.2). Fluo-3-AM-loaded sperm (NC and CAP) were placed on a coverslip coated with 0.03% poly-l-lysine. Subsequently, the sperm were placed in recording chambers, observed and recorded on an Olympus IX71 epifluorescence microscope, using an excitation filter (D485/50X) and an emission filter (HQ535/50 mol l^−1^), with a 465 nm LED as light source (Cairn Research). The setup was coupled to a LED controller, which allowed us to give lighting pulses for an exposure time of 15 ms, taking one image per second. A CCD camera (Andor Technology) was used to record the images. The stimulation protocol consisted of the micropipette addition of the following compounds: P4 (300 nmol l^−1^, 1 µmol l^−1^ and 3 µmol l^−1^), ionomycin (5 µmol l^−1^), MnCl_2_ (5 mmol l^−1^), NH_4_Cl (10 mmol l^−1^) and RU1968 (CatSper-specific inhibitor) (5 µmol l^−1^) (Chávez et al., 2014; Mata-Martínez et al., 2018; Rennhack et al., 2018).

Fluorescence images for Ca^2+^ were analyzed with Andor IQ2 software and ImageJ 1.52 (National Institutes of Health), selecting the head as the region of interest in each sperm. The fluorescence intensity values were normalized using the following equation:
(1)

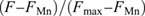


(López-Torres et al., 2017; Mata-Martínez et al., 2018), where *F*_max_ corresponds to the fluorescence obtained after ionomycin addition and *F*_Mn_ refers to the resultant fluorescence after MnCl_2_ addition (MnCl_2_ fluorescence quenching).

### Evaluation of sperm motility

After capacitation, cells were divided into two aliquots, with the concentration adjusted to 10×10^6^ sperm ml^−1^, centrifuged (300 ***g***, 5 min) and resuspended in BWW medium at pH 7.2 (tube 1) and pH 8.0 (tube 2). In each sample, motility was stimulated with 4-aminopyridine (4-AP; weak base that increases pH and thus stimulates CatSper) (Achikanu et al., 2018) or treated with 5 µmol l^−1^ RU1968 (Rennhack et al., 2018). Five microliters of the sperm samples for each condition were loaded into a Spermatrack Proiser chamber at 37°C and immediately placed on the heated stage (37°C) of a Nikon model ECLIPSE Ci-L upright microscope with an attached computer-assisted sperm analysis (CASA) system equipped with a Basler Ace camera (version 5.4, Microptic) and the Sperm Class Analyzer 5 Software (López-Torres et al., 2017; World Health Organization, 2010). The percentage motility was determined (progressive, non-progressive and static), in addition to the kinematic parameters, without the addition of stimuli and 300 s after their application. Approximately ten video photographs were collected, in which at least 300 sperm were tracked (up to 15 fields). The criteria for determining hyperactivation were curvilinear velocity (VCL)≥150 μm s^−1^, linearity (LIN)<50% and amplitude of lateral head movement (ALH)≥3.5 μm (Mortimer and Mortimer, 2013).

### Evaluation of the membrane potential

Samples of 10×10^6^ sperm ml^−1^ (NC and CAP) were incubated (37°C, 5 min) with DISC3-(5) dye (1 µmol l^−1^). Subsequently, the samples were incubated with the mitochondrial uncoupler CCCP (500 nmol l^−1^) for 2 min. Valinomycin (1 µmol l^−1^) was added, followed by sequential additions of KCl (7.5, 12.5, 22.5 and 42.2 mmol l^−1^). The additions of CCCP (mitochondrial uncoupler), valinomycin and KCl were performed as part of the calibration method to determine the resting membrane potential (*E*_m_) of the cells in the different experimental conditions. The fluorescence of the cells was recorded in each condition (Chávez et al., 2013). The *E*_m_ measurements were taken using an Aminco SLM spectrofluorometer, operated by Olis software (Bogart, GA, USA). For the excitation of DISC3-(5) dye, a 640 nm LED was used with an excitation/emission filter system of 640/670 nm, respectively (Chroma Technology). *E*_m_ theoretical values were calculated using the Nernst equation, assuming that the intracellular K^+^ concentration ([K^+^]_i_) was 120 mmol l^−1^, as determined by Babcock (1983) in bovine sperm. The final *E*_m_ values were obtained by interpolating the theoretical values with the arbitrary fluorescence units (AFU) recorded from each trace (Chávez et al., 2013).

### Electrophysiological recordings of bat sperm

For this technique, we used epididymal sperm with a visible cytoplasmic droplet. A 50 µl aliquot was placed onto untreated glass coverslips of a recording chamber at room temperature (22°C). Non-motile sperm were washed repeatedly with a physiological solution, HS pH 7.4 (with NaOH). The electrical seal between the patch pipette and the membrane cell was formed in the cytoplasmic droplet. Recording in monovalent solution was done for CatSper currents in whole-cell mode. The pipette solution contained 135 mmol l^−1^ Cs-methanesulphonate, 5 mmol l^−1^ CsCl, 10 mmol l^−1^ EGTA, 5 mmol l^−1^ Na_2_ATP, 10 mmol l^−1^ HEPES, pH 7.0 (with CsOH). The different compounds used to alter the function (gain or loss) of the CatSper channel were added directly to the recording chamber. A horizontal puller (P-2000 Sutter Instrument Co., Novato, CA, USA) was used to make the pipettes, and the tip was heat polished using a micro-forge (Narishige MF-83, Scientific Instrument Lab, Setagaya-Ku, Tokyo, Japan). The electrical resistance of the pipettes after filling them with pipette solution was 10–15 MΩ. The cells were stimulated by applying a standard voltage-ramp protocol from −80 mV to +80 mV with holding potential of 0 mV every 5 s using an Axopatch 200 amplifier (Molecular Devices, Sunnyvale, CA, USA) connected to an analog-to-digital converter DigiData 1200 (Molecular Devices). The data were collected using pClamp 6 software (Molecular Devices) and analyzed offline with Clampfit 10.7 (Molecular Devices) and SigmaPlot 10.0 (Systat Software, Inc. San Jose, CA, USA) software.

### Data analysis

The results are presented as mean±s.e.m., determining significant differences between the groups using one-way ANOVA (*P*<0.05), Tukey–Kramer *post hoc*. Testicular and epididymal weights, sperm concentration, sperm motility, and *E*_m_ and hyperactivation for every month of capture were included in a data comparison matrix. Electrophysiology results were analyzed with a paired two-tailed Student’s *t*-test (*P*<0.01). Statistical analysis was performed with the statistical package R Studio Version 1.2.5033 (https://www.npackd.org/p/rstudio/1.2.5033).

## RESULTS

It was previously reported that sperm stored in the caudal region of the epididymis of *C. mexicanus* maintain their fertile capacity during a prolonged storage period (Rodríguez-Tobón et al., 2016), even when the testicles involute once the sperm are released from the seminiferous tubules and enter the epididymis. In this study, testicular mass in *C. mexicanus* decreased gradually from the cessation of spermatogenesis (September, 0.076±0.0021 g) until the copulation period (November, 0.0162±0.0018 g). We determined that the slight decrease in epididymal mass from September (0.111±0.0093 g) to November (∼0.07±0.0084 g) could be related to the number of sperm that can be found in the epididymal cauda of *C. mexicanus* (Fig. S1A). Previously, it was reported that *C. mexicanus* whole-body mass in September, October and November does not show considerable variation, because values of 7.7±0.2 g, 7.3±0.2 g and 7.1±0.1 g, respectively, were obtained in these months (León-Galván et al., 2005). In this study, sperm concentration was determined after the swim-out, and the highest number of cells was observed in October (156×10^6^±7 sperm ml^−1^) in comparison with September (108×10^6^±15.5 sperm ml^−1^) and November (101×10^6^±6.18 sperm ml^−1^) (Fig. S1B). To establish the physiological state of the gametes, the percentage motility was analyzed during the months of sperm storage (September to November). In this period, the motility was ∼85% for all the months studied (Fig. S1C).

### Analysis of sperm motility patterns by CASA

**Fig. 1.**
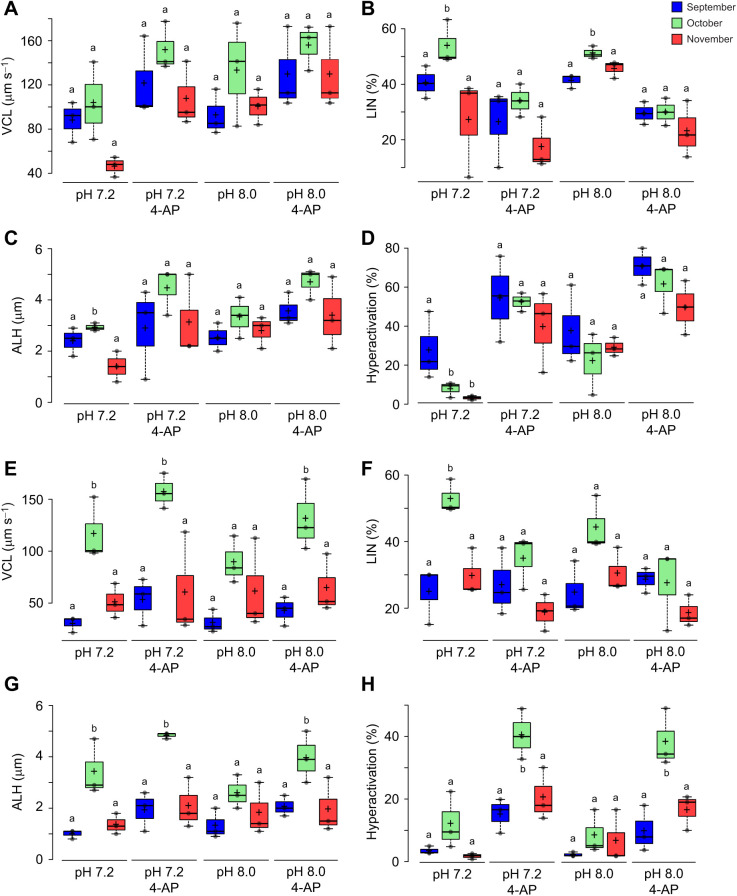
**Hyperactivation of *Corynorhinus mexicanus* sperm, activated with 4-AP and alkaline pH.** (A–H) Evaluation of kinematic parameters and hyperactivation in epididymal sperm under NC (A–D) and CAP (E–H) conditions: curvilinear velocity (VCL; A,E), linearity (LIN; B,F), amplitude of lateral head movement (ALH; C,G) and hyperactivated motility (D,H). Different letters indicate statistically significant differences (*P*<0.05) when comparing the months of capture between pH conditions (pH 7.2, pH 7.2+4-AP, pH 8 and pH 8+4-AP) using ANOVA followed by a Tukey–Kramer *post hoc* test. In each box, center lines show the medians; box limits indicate the 25th and 75th percentiles as determined by R software. Whiskers extend to the interquartile range from the 25th and 75th percentiles crosses represent sample means, and data points are plotted as gray circles. *n*=3. 4-AP, 4-aminopyridine.

For a detailed analysis of sperm motility, progressive, non-progressive, and static or immotile sperm were evaluated (Fig. S2). No variation was observed in the percentage of static sperm among the months of capture. A statistically significant decrease was found in progressive motility in November (37%) compared with September (58%) and October (54%). Non-progressive motility increased towards November, with a difference of ∼20% compared with September and October (Fig. S2). Considering the importance of pH in sperm physiology and its variation in the female reproductive tract, we evaluated hyperactivation at two pH values (7.2 and 8.0). Fig. 1 shows the kinematic parameters of sperm under NC (Fig. 1A–D) and CAP (Fig. 1E–H) conditions at pH 7.2. and 8.0. Hyperactivation was determined considering the following: VCL≥150 μm s^−1^, LIN<50% and ALH≥3.5 μm. Hyperactivated motility is characterized by an increase in VCL and ALH and a decrease in LIN. In NC conditions, we detected an increase in VCL and ALH during stimulation with 4-AP (Fig. 1A,C), as well as a significant decrease in LIN at pH 8.0 (Fig. 1B). Therefore, the highest percentage of sperm with hyperactive motility was recorded at pH 8.0, without significant differences among the months of capture of *C. mexicanus* (Fig. 1D). In CAP conditions, we observed that the maximum values for VCL and ALH were in October at pH 7.2 and 8.0 in the presence of 4-AP (Fig. 1D,F). In the three months of capture in the absence and presence of 4-AP, there were no statistically significant differences in LIN (Fig. 1E). However, 4-AP induced a significant increase in hyperactivation in samples obtained in October (pH 7.2 and 8.0) and November (pH 7.2) (Fig. 1H). In the absence of 4-AP, the highest percentage of hyperactivation was recorded in samples obtained in October (12% at pH 7.2 and 9% at pH 8.0). Surprisingly, CAP conditions at both pHs did not produce an increase in hyperactivated sperm motility, when compared with NC conditions.

### Evaluation of biochemical indicators in sperm capacitation

#### Single cell [Ca^2+^]_i_ assays

Based on the importance of Ca^2+^ in the capacitation process, we analyzed [Ca^2+^]_i_ in NC and CAP conditions. CatSper is a Ca^2+^-selective channel located in the principal piece in sperm flagella. However, its presence in *C. mexicanus* is unknown despite expression in all mammals in which it has been evaluated. Therefore, to determine the presence of CatSper in this species, we analyzed the sensitivity of sperm to P4 and alkalinization (using NH_4_Cl) as activators of this channel.

**Fig. 2.**
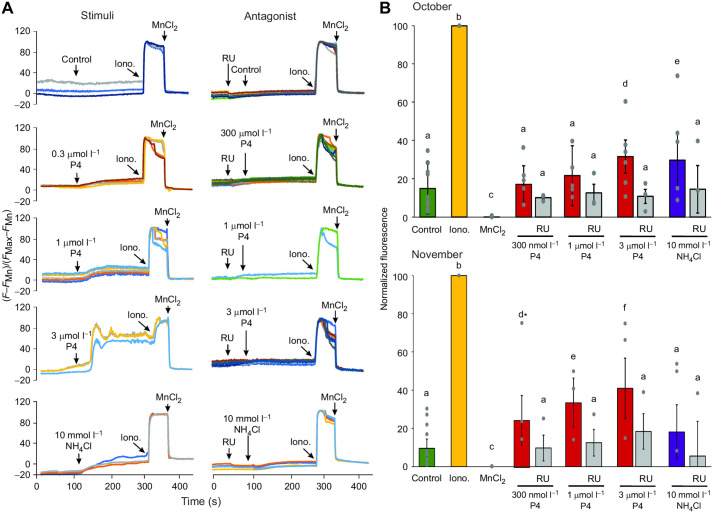
**P4 (3 µmol l^−1^) and NH_4_Cl induce a significant increase in Ca^2+^ in NC sperm.** Fluo-3-AM-loaded *C. mexicanus* sperm in single-cell recordings. (A) Representative recordings of the Ca^2+^ dynamics stimulated with different P4 concentrations and NH_4_Cl without (left) and with (right) CatSper antagonist RU1968. Each line represents different cells; the stimuli are indicated by arrows. After the stimuli, we added ionomycin (5 µmol l^−1^) and MnCl_2_ (5 mmol l^−1^) to normalize the responses. (B) Summarized data from A, with normalization with respect to ionomycin (100%) and MnCl_2_ (0%). Different letters indicate statistically significant differences (*P*<0.05) under different P4 concentrations, using one-way ANOVA followed by a Tukey–Kramer *post hoc* test. *Significant difference when comparing the different concentrations of P4. The bars indicate mean±s.e.m., and individual data points are plotted as gray circles. *N*=3 per month. Control, BWW medium addition (green bars); Iono., ionomycin (yellow bars; 5 µmol l^−1^); MnCl_2_, magnesium chloride (black bars; 5 mmol l^−1^); NC, non-capacitated; NH_4_Cl, ammonium chloride (blue bars; 10 mmol l^−1^); P4, progesterone (red bars, 300 nmol l^−1^, 1 µmol l^−1^ and 3 µmol l^−1^); RU, RU1968 (gray bars; 5 µmol l^−1^).

**Fig. 3.**
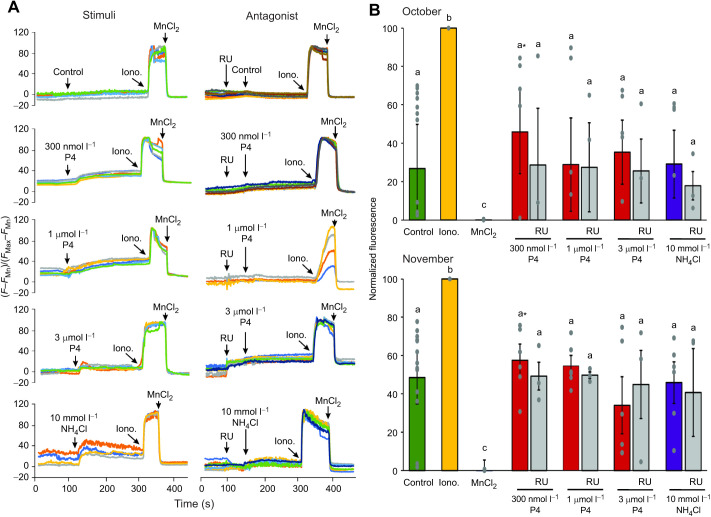
**P4 and NH_4_Cl induce a non-significant increase in Ca^2+^ in CAP sperm.** Fluo-3-AM-loaded *C. mexicanus* sperm in single-cell recordings. (A) Representative recordings of the Ca^2+^ dynamics stimulated with different P4 concentrations and NH_4_Cl without (left) and with (right) CatSper antagonist RU1968. Each line represents different cells; the stimuli are indicated by arrows. After the stimuli, IONO (5 µmol l^−1^) and MnCl_2_ (5 mmol l^−1^) were added to normalize the responses. (B) Summarized data from A, using a double normalization with ionomycin as 100% and MnCl_2_ as 0%. Different letters indicate statistically significant differences (*P*<0.05) under different P4 concentrations, using one-way ANOVA followed by a Tukey–Kramer *post hoc* test. *Significant difference when comparing the different concentrations of P4. The bars indicate the mean±s.e.m., and individual data points are plotted as gray circles. *N*=3 per month. CAP, capacitated; Control, BWW medium addition (green bars); Iono., ionomycin (yellow bars; 5 µmol l^−1^); MnCl_2_, magnesium chloride (black bars; 5 mmol l^−1^); NH_4_Cl, ammonium chloride (blue bars; 10 mmol l^−1^); P4, progesterone (red bars, 300 nmol l^−1^, 1 µmol l^−1^ and 3 µmol l^−1^); RU, RU1968 (gray bars; 5 µmol l^−1^).

Ca^2+^ dynamics were recorded before and after the addition of P4 (0.3 µmol l^−1^, 1 µmol l^−1^ and 3 µmol l^−1^) or 10 mmol l^−1^ NH_4_Cl (Figs 2 and 3, Fig. S3) in single-cell fluorescence imaging experiments. We observed that P4 significantly increased [Ca^2+^]_i_ compared with the control (addition of medium without P4) in sperm under NC (Fig. 2) and CAP (Fig. 3) conditions. Consistently with the presence of CatSper, preincubation with RU1968 inhibited both P4 (0.3 µmol l^−1^, 1 µmol l^−1^ and 3 µmol l^−1^) and NH_4_Cl responses in NC conditions (Fig. 2). Additionally, we compared the P4- and NH_4_Cl-induced [Ca^2+^]_i_ increase in sperm samples obtained in October and November. In NC sperm, the [Ca^2+^]_i_ increase in the three P4 concentrations was slightly higher in samples from November compared with those from October (orange, blue and red bars in Fig. 2B). However, there was a decrease in the Ca^2+^ response with NH_4_Cl stimulation in samples from November compared with those from October (purple bars in Fig. 2B). The CatSper antagonist, RU1968 (gray bars in Fig. 2B), caused a significant reduction in the [Ca^2+^]_i_ increase induced by P4 and NH_4_Cl in NC (Fig. 2) conditions. In CAP conditions (Fig. 3), we observed a stronger response to P4 and NH_4_Cl compared with that in NC conditions. We also observed an increase in the Ca^2+^ response to P4 and NH_4_Cl stimuli in samples from November compared with those from October. We observed a slight, but not significant, decrease in the [Ca^2+^]_i_ in response to P4 and NH_4_Cl in the presence of RU1968 in CAP conditions (Fig. 3).

Owing to experimental adjustments and restrictions for bat capture, we were unable to collect sufficient data in September. Nevertheless, we believe that data from October and November provide a good representation of the behavior of the population.

### Electrophysiological recordings of sperm

**Fig. 4.**
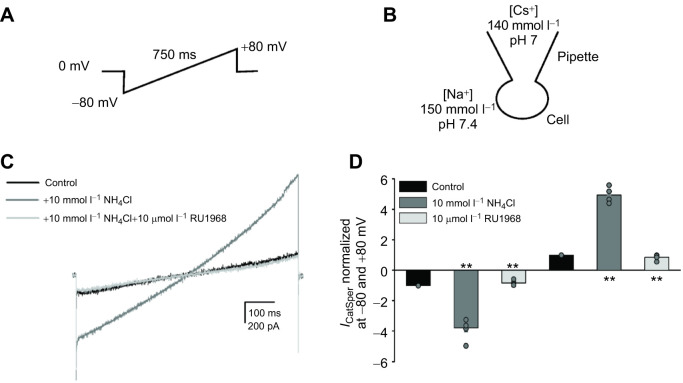
**Whole-cell patch-clamp recordings *of C. mexicanus* sperm, showing CatSper-like monovalent cationic currents stimulated by NH_4_Cl and inhibited by RU1968.** (A) Standard voltage-ramp protocol used to elicit the macroscopic currents of CatSper. (B) Graphic representation of the whole-cell mode and solutions by monovalent current recordings. (C) A family of currents elicited in a representative spermatozoon by the voltage protocol applied in A under the following conditions: control (black trace), alkalization with NH_4_Cl (dark-gray trace) and in the presence of the CatSper inhibitor RU1968 (light-gray trace). (D) Summary of results obtained from five different bats under the above experimental conditions (control, black bars; NH_4_Cl, dark-gray bars; RU1968, light-gray bars). The data were normalized with respect to control and presented as s.e.m. and individual data points are plotted as gray circles. Statistical significance was determined using paired two-tailed Student’s *t*-test. (**P<0.01). *N*=5.

The sperm-specific Ca^2+^ channel (CatSper) is activated by P4 and alkalinization in human sperm. To investigate the presence of this channel in *C. mexicanus*, we performed patch-clamp electrophysiological recordings in male gametes of *C. mexicanus* (Fig. 4). We used the whole-cell configuration under DVF conditions, applying a ramp starting at 0 mV up to −80 mV and to +80 mV, with 10 mV pulses (Fig. 4A,B). Our recordings suggest the presence of CatSper in sperm from *C. mexicanus* because we observed currents (*I*_CatSper_) that were upregulated by NH_4_Cl (Fig. 4C). Activation by NH_4_Cl was blocked by the CatSper-specific inhibitor RU1968 (Fig. 4C), resulting in significant differences compared with the control (Fig. 4D).

### Plasma *E*_m_ measurements

**Fig. 5.**
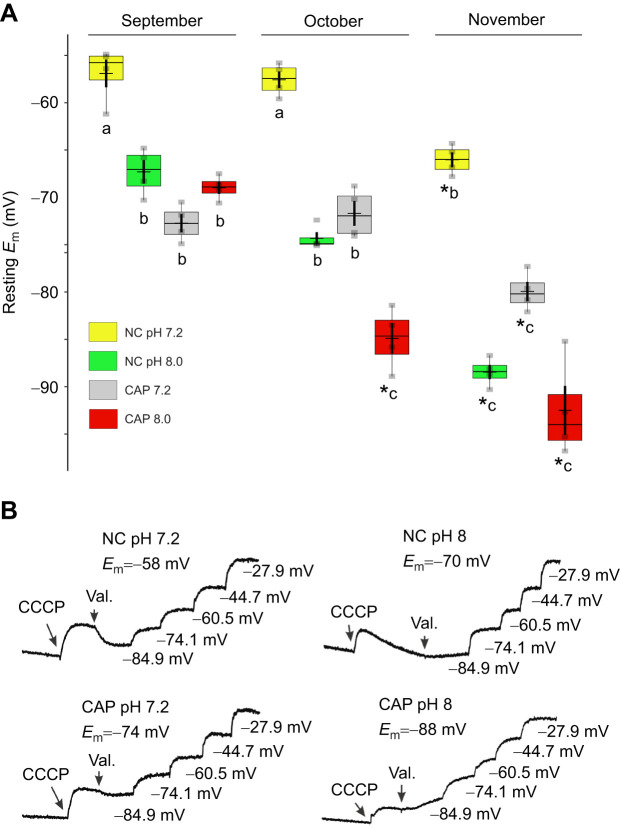
**Capacitation and alkalinization of the extracellular medium induce plasma membrane hyperpolarization in *C. mexicanus* sperm.** (A) Membrane potential (*E*_m_) determinations using the fluorescent dye DiSC3-(5) under different experimental conditions: non-capacitated (NC) pH 7.2 (yellow) and pH 8.0 (green), and capacitated (CAP) pH 7.2 (gray) and pH 8.0 (red), during the months of capture. (B) Representative traces of sperm from a bat captured in October, showing the calibration obtained by adding a mitochondrial uncoupler carbonyl cyanide chlorophenylhydrazone (CCCP, first arrows) and valinomycin (Val., second arrows), followed by sequential additions of K^+^. *P≤0.05 using Mann–Whitney Wilcoxon test. In each box, center lines show the medians; box limits indicate the 25th and 75th percentiles as determined by R software. Whiskers extend to the interquartile range from the 25th and 75th percentiles, crosses represent sample means, and data points are plotted as gray rectangles. *N*=3.

During sperm capacitation, *E*_m_ hyperpolarization has been observed in human (López-González et al., 2014), mouse (Santi et al., 2010), bull (Zeng et al., 1995), horse (McPartlin et al., 2011) and hamster (Takei and Kon, 2021), but not in bats. Therefore, we decided to explore whether there is a change in *E*_m_ during capacitation in *C. mexicanus* sperm in cell population experiments and whether there is a difference correlated with epididymal storage. *E*_m_ values were determined under NC and CAP conditions at pH 7.2 and 8.0 in sperm samples of *C. mexicanus* captured during September, October and November. In NC conditions at pH 7.2, we obtained values of −56.9 mV (September), −57.6 mV (October) and −66.0 mV (November). In NC conditions at pH 8.0, we observed hyperpolarization of the *E*_m_ in September (−72.7 mV), October (−71.7 mV) and November (−79.9 mV). In CAP conditions, we observed *E*_m_ hyperpolarization with statistically significant differences at pH 7.2 in all the months of capture, with values of −67.3 mV in September, −74.3 mV in October and −88.4 mV in November. Finally, we observed the highest hyperpolarization in CAP conditions at pH 8.0, with values of −68.9 mV in September, −84.9 mV in October and −92.5 mV in November (Table 1 and Fig. 5A).

**Table 1.**
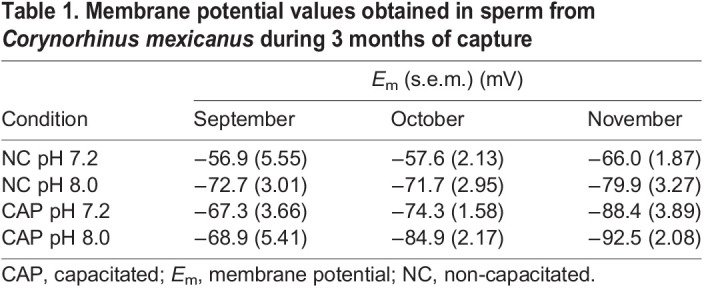
**Membrane potential values obtained in sperm from *Corynorhinus mexicanus* during 3** **months of capture**

## DISCUSSION

*Corynorhinus mexicanus* presents an asynchronous seasonal monoestrous reproductive pattern (León-Galván et al., 2005; López-Wilchis, 1989). The male presents a temporal asynchrony between its primary and secondary reproductive functions (León-Galván et al., 2005). That is, spermatogenesis occurs in summer, which not only implies involution of the testes during autumn, but also a possible decrease in testosterone (León-Galván et al., 2005). However, although the epididymis is an androgen-dependent organ, it has already been shown that the sperm are in good condition during the prolonged storage period (∼5 months, September to January) in the epididymis (Cervantes et al., 2008; Rodríguez-Tobón et al., 2016). In this study, a gradual involution of the epididymal mass was observed during the months of sperm storage, which could be related to the beginning of the copulation period (Crichton y Krutzsch, 2000; León-Galván et al., 2005).

During their transit through the epididymis, sperm undergo a process recognized as epididymal sperm maturation (Gervasi y Visconti, 2017). It has been considered that the increase in the percentage of motile sperm in epididymal sperm is an indicator of maturation (Rodríguez-Tobón et al., 2016). Previous motility studies, based on microscopic procedures reported in the World Health Organization (2010) manual, have shown that sperm stored in the cauda epididymis of *C. mexicanus* maintain more than 80% total motility during September and October. Furthermore, these sperm are capable of undergoing capacitation (measured with CTC procedure) and acrosomal reaction (using the Coomassie Brilliant Blue method) *in vitro* after several months of storage (Rodríguez-Tobón et al., 2016; Cervantes et al., 2008). In addition, Wang et al. (2008) indicated that the sperm progressive motility in *Myotis ricketti* analyzed by CASA is not affected during prolonged sperm storage. Like in *M. ricketti* sperm, the percentage of progressive motility observed in the male gametes of *C. mexicanus* (Fig. S2), even after the involution of the testicle (Fig. S1), is not affected, a phenomenon that is not observed in most mammalian species, because the functionality of the epididymis depends on the concentrations of testosterone produced by the male gonad (Dr Miguel Galván, Universidad Autónoma Metropolitana, personal communication).

To analyze hyperactivated sperm motility in *C. mexicanus*, we examined kinematic motility parameters using a CASA system. We considered the parameters described in other mammals, in terms that hyperactivation is characterized by an increase in VCL and ALH and a decrease in LIN. Cervantes et al. (2008) analyzed *in vitro* capacitation of *C. mexicanus* sperm using fluorescence patterns with CTC, determining that the highest percentage of CAP sperm occurs in October, similar to results reported by Rodríguez-Tobón et al. (2016). Consistently, we observed an increase in VCL and ALH in October, which suggests that sperm require at least 1[Supplementary-material sup1]month of storage in the epididymis to reach their maximum state of maturation. However, in *C. mexicanus*, we observed that sperm incubated under CAP conditions did not show an increase in hyperactivated motility (Fig. 1D) at pH 7.2. Additional experiments will be necessary to explore the period in which maximum hyperactivation occurs. It is important to note the limited literature available for *C. mexicanus* sperm and that we therefore based our determination of hyperactivation on the parameters established for human sperm, owing to similarities in the length of the head and the flagella. Additional studies/analyses are required to establish the optimal hyperactivation parameters for bat sperm. However, incubation at alkaline pH (pH 8.0) increases the percentage of hyperactivation under NC and CAP conditions, compared with that at pH 7.2 (Fig. 1D,H), suggesting that the entity responsible for hyperactivation in bat sperm is sensitive to alkalinization, probably CatSper (Stival et al., 2016; Suarez and Ho, 2003). In addition, sperm incubated with 4-AP showed a significant increase in this motility pattern (Fig. 1D). It has been reported that the addition of 4-AP raises pH, activates CatSper and therefore stimulates hyperactivation (Achikanu et al., 2018; Ardon et al., 2016; Gu et al., 2004).

CatSper has been found in a wide spectrum of phylogenetic groups. Romero and Nishigaki (2019) reported the presence of CatSper in mammals, reptiles, coelacanths, cartilaginous fish, amphioxus tunicates, echinoderms, brachiopods, cnidarians and ctenophores (Rahban and Nef, 2020; Singh and Rajender, 2015). However, the presence of this channel in the order Chiroptera was not specified. To demonstrate the presence of CatSper in *C. mexicanus* sperm, we performed [Ca^2+^]_i_ measurements under stimulation by P4 and alkalinization, two well-accepted activators of CatSper. In addition, we previously showed that a concentration of 3 µmol l^−1^ of P4 increases the acrosome reaction in sperm obtained during prolonged storage and subjected to *in vitro* capacitation (Cervantes et al., 2008).

In this work, we observed an increase in [Ca^2+^]_i_ in a concentration-dependent manner from 0.3 to 3 µmol l^−1^ P4, and with 10 mmol l^−1^ NH_4_Cl. This response was partially inhibited by the CatSper blocker RU1968. We observed that, under NC conditions, the highest magnitude of response to P4 was obtained at 3 µmol l^−1^. Under CAP conditions, 0.3 µmol l^−1^ P4 provoked the highest transient entry of Ca^2+^. These data indicate that, in *C. mexicanus*, the sperm are sensitive to low P4 concentrations, such as human sperm observed in Ca^2+^ measurement studies (Harper et al., 2003; Strünker et al., 2011; Servin-Vences et al., 2012), in which it was found that 500 nmol l^−1^ P4 activates CatSper. Our results provide new evidence for answering evolutionary questions about CatSper activation in mammals. Interestingly, P4 appears not to be the physiological activator of CatSper in bovine, porcine and mouse spermatozoa because concentrations of 0.1–10 µmol l^−1^ do not activate CatSper in these species (Miguel-Jiménez et al., 2020). In mouse spermatozoa, concentrations of 50–100 µmol l^−1^ are required to activate CatSper (Lishko et al., 2011; Romarowski et al., 2016). These findings indicate that, in this respect, chiropteran spermatozoa are more similar to human spermatozoa than to mouse spermatozoa, although they are not phylogenetically very close (Tsagkogeorga et al., 2013).

Regarding the storage time in epididymis related to the months of capture, we observed a slight increase in [Ca^2+^]_i_ stimulated with P4 in sperm collected in November compared with those collected in October in NC conditions, but no differences were found in CAP conditions in the same periods. Further experiments are required to obtain conclusive results, because in CAP conditions P4 stimulation was stronger than in NC conditions, and the effect of the CatSper antagonist was lower.

To confirm the presence of CatSper in *C. mexicanus* sperm, we performed electrophysiological recordings in DVF conditions using a voltage-ramp stimulation, under control conditions and in the presence of NH_4_Cl. We observed currents activated upon alkalinization that were inhibited by RU1968, and therefore it is likely that CatSper channels are present and functionally active in *C. mexicanus* sperm. However, owing to the partial inhibition of RU1968 under P4 and NH_4_Cl stimuli in Ca^2+^ measurements, we do not disregard the presence of additional Ca^2+^ entities activated by P4 or intracellular alkalinization. The variations observed with the CatSper inhibitor RU1968 (Rennhack et al., 2018) may be caused by differences in its activity, or the site of interaction between the inhibitor and *C. mexicanus* CatSper. Also, Rennhack et al. (2018) described that the potency of RU1968 to inhibit CatSper by alkalinization decreases with increasing intracellular pH (pH_i_) in human sperm.

The analysis of *E*_m_ in *C. mexicanus* sperm revealed hyperpolarization in CAP compared with NC conditions at pH 7.2, which increased according of the month of capture, displaying a higher hyperpolarization in November compared with September and October. Moreover, bat sperm incubation in alkaline conditions (pH 8.0) increased the hyperpolarization values, with higher *E*_m_ negative values obtained in November. It is necessary to point out that the values were calculated taking into account a [K^+^]_i_ estimated in other species (Babcock, 1983). Therefore, the values are not precise for *C. mexicanus*, but we believe that the trends are reliable. In terms of the molecular entity responsible for the bat sperm hyperpolarization, previous reports in mouse and human sperm described the presence of K^+^ channels from the SLO family (Chávez et al., 2013; Puga-Molina et al., 2020; Santi et al., 2010; López-González et al., 2014). We observed hyperpolarization under sperm incubation at alkaline pH, suggesting the presence of a pH-sensitive channel. Further experiments should be done to determine the specific molecular entity, but our results allow us to propose two hypotheses: (1) in sperm from *C. mexicanus*, the SLO3 channel may be present; and (2) the activity of this channel could be the regulator of the hyperpolarization of these cells under CAP conditions, owing to the increase in hyperpolarization that we observed at pH 8.0.

In summary, our results indicate that (1) *C. mexicanus* sperm exhibit significant hyperpolarization of the *E*_m_ under CAP conditions; (2) *C. mexicanus* NC sperm have more negative *E*_m_ values compared with other mammals such as humans and mouse; (3) the sensitivity of sperm to P4, NH_4_Cl and RU1968 suggest the presence of CatSper in *C. mexicanus* sperm; and (4) the absence of hyperactivated motility in CAP conditions in *C. mexicanus* sperm is not conclusive because the kinematic characteristics of motility have not been described in bat. Moreover, hyperpolarization and P4 response through CatSper activation increased according to the time of epididymal storage, indicating that *C. mexicanus* undergoes capacitation processes similar to those in other mammals and also that the biochemical elements must be more active or prepared to be activated once the mating season begins.

## Supplementary Material

10.1242/jexbio.244878_sup1 Supplementary information
