# Supplementary material for: Sperm calcium flux and membrane potential hyperpolarization observed in the Mexican big-eared bat Corynorhinus mexicanus
Source: J Exp Biol. 2023 Jan 30;226(2):jeb244878. doi: 10.1242/jeb.244878 (PMC10086540; doi:10.1242/jeb.244878)
Supplement: Supplementary information [file jexbio-226-244878-s1.pdf]

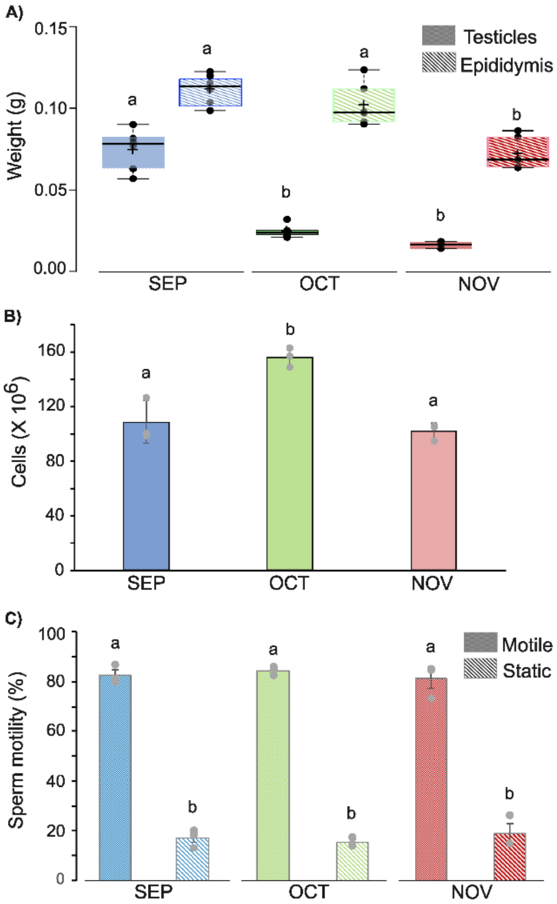

Fig. S1. There is a testicles involution due to sperm storage in epididymis. Sperm motility is conserved in epididymis during prolonged storage in *Corynorhinus mexicanus*. A) Testicular (solid boxes) and epididymal (diagonal lines boxes) weight in the different moths of capture: September (SEP) (blue boxes), October (OCT) (green boxes) and November (NOV) (red boxes). In each box, center lines show the medians, box limits indicate the 25th and 75th percentiles as determined by R software. Outliers are represented by dots, crosses represent sample means and data points are plotted as small black circles. **B)** Concentration of sperm (cells x 10^6) obtained from the caudal epididymal region, during the different moths of capture: September (SEP) (blue bar), October (OCT) (green bar) and November (NOV) (red bar). **C)** Percentage of motile sperm obtained from the epididymal cauda. solid bars: motile sperm and diagonal lines bars: Static sperm. The bars in **B)** and **C)** indicate the means ± S.E.M., and data points are plotted as small gray circles. n=3 per month. Different letters indicate statistically significant differences (p<0.05) when compared between the different capture months.

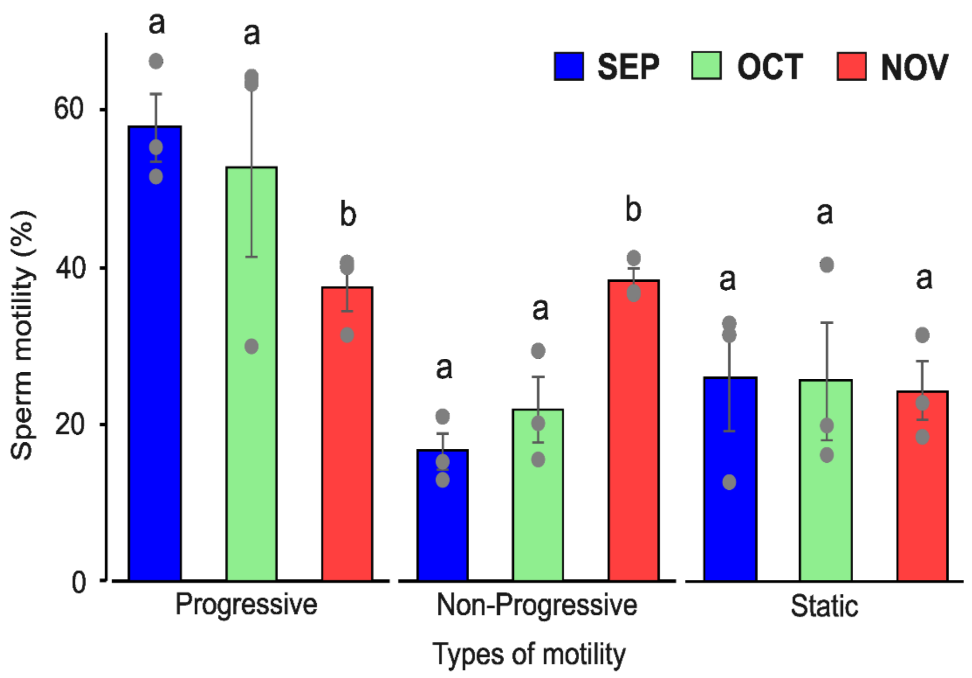

Fig. S2. Progressive and non-progressive sperm motility parameters are statistically different in November compared to September and October. Comparison of the percentage of sperm motility in three types of parameters: progressive, non-progressive and static, during the indicated months of capture. The bars indicate the means ± S.E.M., and data points are plotted as small gray circles. n=3 per month. Different letters indicate statistically significant differences (p <0.05) when comparing each type of motility between the different months of capture, using ANOVA followed by a Tukey-Kramer post hoc test. SEP: September (blue bars), OCT: October (green bars) and NOV: November (red bars).

Journal of Experimental Biology • Supplementary information

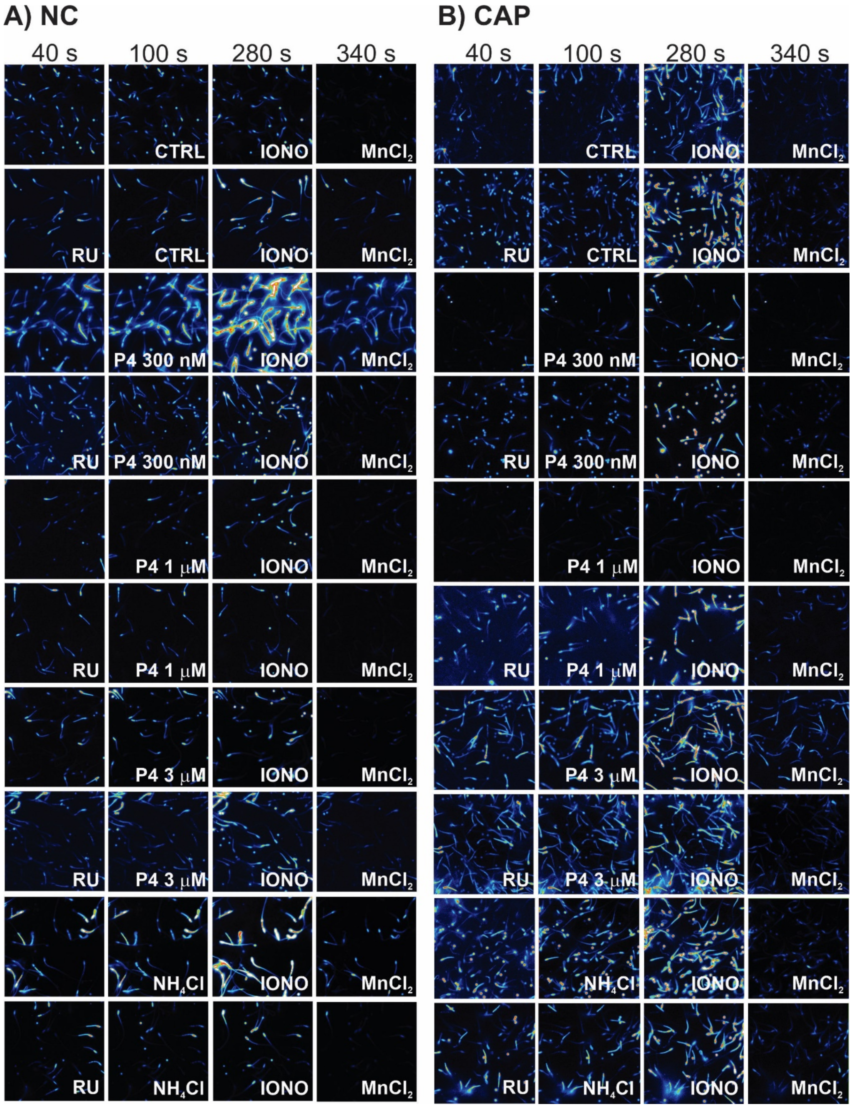

Fig. S3. P4 and NH4Cl increases [Ca^{2+}]i in *C. mexicanus* bat sperm, under NC and CAP conditions. This response is inhibited by the CatSper blocker RU1968. A) NC time-lapse frames at the indicated times (40 s, 100 s, 280 s and 340 s) of sperm in NC conditions. The addition is shown in each frame (lower right corner: RU, CTRL, P4, NH4Cl, IONO and MnCl2). **B)** time-lapse frames at the indicated times (60 s, 100 s, 280 s and 340 s) of sperm in CAP conditions. The additions are shown in each frame (lower right corner: RU, CTRL, P4, NH4Cl, IONO and MnCl2). RU= RU1968 CatSper antagonist (5 μM). CTRL= BWW medium addition. P4= Progesterone (μM 0.3, 1, 3). NH4Cl= Ammonium chloride 10 mM. IONO= Ionomycin 5 μM. MnCl2= Manganese chloride 5 mM.

Journal of Experimental Biology • Supplementary information
